# The influence of renal function on the association of rs854560 polymorphism of paraoxonase 1 gene with long-term prognosis in patients after myocardial infarction

**DOI:** 10.1007/s00380-014-0574-8

**Published:** 2014-08-26

**Authors:** Anna Szpakowicz, Witold Pepinski, Ewa Waszkiewicz, Dominika Maciorkowska, Małgorzata Skawronska, Anna Niemcunowicz-Janica, Sławomir Dobrzycki, Włodzimierz J. Musial, Karol A. Kaminski

**Affiliations:** 1 Department of Cardiology, Medical University of Bialystok, M. Sklodowskiej-Curie 24a, 15-276 Bialystok, Poland; 2 Department of Forensic Medicine, Medical University of Bialystok, Waszyngtona 13, 15-230 Bialystok, Poland; 3 Department of Invasive Cardiology, Medical University of Bialystok, M. Sklodowskiej-Curie 24a, 15-276 Bialystok, Poland

**Keywords:** Paraoxonase, Myocardial infarction, Prognosis, rs662, rs854560, Gene polymorphism, Renal function

## Abstract

Paraoxonase 1 (PON1) is an enzyme responsible for the antioxidant properties of high density lipoprotein (HDL). The activity of PON1 is decreased in patients with coronary artery disease, myocardial infarction or chronic kidney disease. rs662 and rs854560 are single nucleotide polymorphisms (SNPs) associated with PON1 activity and 10-year cardiovascular mortality of patients with stable coronary artery disease. We investigated the association of rs662 and rs854560 SNPs of the PON1 gene with 5-year mortality in patients with ST-elevation myocardial infarction (STEMI) treated invasively. We analyzed the data of consecutive patients with STEMI treated with primary PCI. Genotyping was performed with the TaqMan method. The analyzed end-point was total 5-year mortality. Additional subgroup analysis was performed for survival of patients depending on their eGFR. The study group comprised 634 patients (mean age 62.3 ± 11.85 years; 25.2 % of women, *n* = 160; PCI successful in 92.3 %, *n* = 585). No clinically relevant differences in baseline characteristics were found between the genotypes. No association between either genotype and 5-year mortality was found: *p* = 0.4 for the rs662 SNP, *p* = 0.73 for the rs854560 one (log-rank test). However, in a subgroup of patients with eGFR below median value (78.6 ml/min/1.73m2) the rs854560 AA homozygotes had a significantly lower probability of survival (*p* = 0.047, log-rank test). The AA genotype of the rs854560 SNPs of the PON1 gene is associated with increased mortality in patients after myocardial infarction in the subpopulation of patients with lowered eGFR. This phenomenon may be explained by potentially lower PON1 activity in kidney disease.

## Introduction

Paraoxonase 1 (PON1) is an enzyme associated with high density lipoprotein (HDL) that is responsible for its antioxidant properties. PON1 hydrolyzes oxidized phospholipids and cholesterol esters [1]. The activity of PON1 was shown to be decreased in patients with coronary artery disease, myocardial infarction or chronic kidney disease [2–5]. In patients with coronary artery disease decreased PON1 activity leads to malfunction of the HDL molecule with subsequent activation of the LOX-1 receptor (an endothelial lectin-like oxidized LDL-receptor) and endothelial PKCβII, followed by inhibition of eNOS-activating pathways and decreased NO production [6]. In this way, the dysfunctional HDL of impaired function fails to stimulate endothelial repair.

The other factors that influence PON1 activity are rs662 (Q192R) and rs854560 (L55M) single nucleotide polymorphisms (SNPs), [7]. The rs662 SNP leads to transition between adenine and guanine nucleobases that results in glutamine-to-arginine substitution at codon 192 (Q192R). In the case of rs854560 SNP thymine to adenine transversion leads to leucine/methionine variability at codon 55 (L55M). In both cases we receive a missense mutation. The rs662 SNP is the only one that leads to Q192R substitution and, analogically, only the rs854560 SNP leads to L55M variability in a PON1 gene. Therefore rs662 is a synonym for Q192R polymorphism and rs854560 is a synonym for an L55M one. The 192Q (AA) and 55M (AA) are variants associated with lower PON1 activity [7]. This also justifies combined analysis of T and G allel carriers, vs AA homozygotes in both SNPs. The rs662 AA genotype was linked with an unfavorable lipid profile [8] and the lowest PON1 activity in patients with chronic kidney disease [9].

There are several case–control studies that investigate the influence of both SNPs on coronary artery disease prevalence. However, a meta-analysis comprising 43 such studies showed no significant association [10]. Reports concerning the link between the rs662 SNP and myocardial infarction are also ambiguous. In the REGICOR study, AA genotype was significantly more frequent in patients with myocardial infarction compared to control group [3]. In contrast, there are reports that the R allele is associated with increased risk of an acute coronary syndrome [11, 12]. On the other hand, the multicenter ECTIM study revealed no link between PON1 polymorphism and myocardial infarction [13].

The link between rs662 and rs854560 SNPs and coronary artery disease remains unclear. In spite of that, it has been proved that they influence 10-year cardiovascular mortality in this group of patients [1]. The high-risk genotype was the AA in both cases (192Q and 55M). This association remained significant after adjustment for lipid parameters and smoking status.

We assumed that a comparable trend could be observed in long-term observation of patients after myocardial infarction. Therefore the aim of our study was to investigate the association of rs662 and rs854560 SNPs of the PON1 gene with 5-year overall mortality in patients with ST-elevation myocardial infarction (STEMI).

## Materials and methods

### Material

The study group comprised consecutive patients with STEMI who were hospitalized in the years 2001–2005 and survived their first 48 h after admission. All of them were Caucasian, inhabitants of North-Eastern Poland. Early-deceased individuals were excluded from the study, because in their case genetic testing would have no potential role to play in clinical decision making. No other exclusion criteria were implemented. Coronary angiography was performed within 12 h from symptoms onset in all patients. STEMI was diagnosed based on a rise in troponin I concentrations or creatine kinase–MB fraction activity accompanied by chest pain history and new ECG abnormalities (lasting >20 min ST-segment elevation or left bundle branch block). In the analysis we included data from patients’ history, physical examination on admission, routine laboratory tests, echocardiography, results of coronary angiography and invasive treatment. Modification of diet in renal disease (MDRD) formula was used to estimate creatinine clearance (eGFR) [14]. General risk assessment of each patient was performed with the Grace risk score, as previously described [15]. Pharmacological treatment was consistent with contemporary guidelines.

The control group included 222 adult men and 221 adult women, whose genetic material was collected for paternity testing. Their clinical characteristics were not available. However, we assumed that selection of subjects for paternity testing was random from a clinical point of view. Therefore, such a group should be highly representative for our region in terms of genetic background.

### Laboratory procedures

Laboratory procedures were described previously [16]. Blood samples were collected in EDTA tubes, treated with commercial DNA extraction kit (Blood Mini, A&A Biotechnology) and stored at −20 degrees Celsius. The SNPs were assessed with a TaqMan SNP Genotyping Assay on the ABI 7500 real time PCR platform (Applied Biosystems), according to manufacturer’s instructions. Ten percent of samples were genotyped in duplicates.

### End-point

Five-year all-cause mortality was the analyzed end-point. Data concerning deaths and dates of deaths were retrieved from the local population registry run by a Government Office.

### Statistical analysis

STATISTICA 9.0 software was used for statistical analysis. After testing for distribution with Shapiro–Wilk test, clinical parameters were compared between the genotypes with *χ* ^2^ or Kruskal–Wallis tests, as appropriate. Survival was compared with log-rank test. Univariate and multivariate analyses for 5-year survival were performed with Cox proportional hazards model. Based on analysis of survival curves and mortality rates, a recessive model of penetrance was assumed. Two-sided *p* value <0.05 was considered statistically significant. The biostatistical parameters were calculated using ARLEQUIN v.3.0 software.

### Ethics statement

The study protocol was approved by the Ethics Committee of the Medical University of Bialystok. The study was performed in accordance with the ethical standards laid down in the 1964 Declaration of Helsinki. Informed written consent was obtained from all the subjects prior to their inclusion in the study.

## Results

### Characteristics of the study group and genotyping results

A total of 652 patients were included in our registry. Nine patients were lost to follow-up (1.4 %) and in nine cases genotypes could not be determined due to poor sample quality. For all samples genotyped in duplicate consistent results were obtained. The final study group comprised 634 patients (mean age 62.3 ± 11.85 years; 25.2 % of women, *n* = 160; PCI successful in 92.3 %, *n* = 585).

Genotyping results for both SNPs are presented in Table 1. No significant deviations from the Hardy–Weinberg equilibrium were found in the populations analyzed. There was only weak linkage disequilibrium between the rs662 and rs854560 polymorphisms, (LD = 0.0149, *D*` = 0.08, *r* ^2^ = 0.0052, *p* = 0.01). The specific allele frequencies differ between previous reports: however, our genetic distribution was comparable to European databases (1, 3, 9). There was no significant difference between the study and control groups in haplotype distributions (*p* > 0.05, *χ* ^2^ test).

**Table 1:** Percentages of specific genotypes and associated mortality rates. The difference between the study and control groups was not statistically significant (*p* > 0.05, *χ* ^2^ test)

Polymorphism (risk allele)	rs662 (A?^a^)			rs854560 (A)		
Genotype	AA	AG	GG	AA	AT	TT
Study group (*n* = 634)						
Percentage (*n*)	52.4 (332)	40.7 (258)	6.9 (44)	46.7 (296)	42.4 (269)	10.9 (69)
5-year mortality (*n*)	15.4 (51)	18.6 (48)	11.4 (5)	17.2 (51)	16.4 (44)	13.0 (9)
Control group (*n* = 443)						
Percentage (*n*)	54.8 (243)	39.7 (176)	5.4 (24)	45.4 (201)	45.1 (200)	9.5 (42)

^a^Reports are ambiguous

Tables 2 and 3 show clinical characteristics of the study group based on the rs662 and rs854560 genotypes, respectively. There was a significant difference between rs662 genotypes in the percentages of implanted stents and successful angioplasties.

**Table 2:** Baseline characteristics of the study group based on rs662 genotype

Characteristic	Overall population (*N* = 634)	AA homozygotes (*N* = 332)	Heterozygotes (*N* = 258)	GG homozygotes (*N* = 44)	*p*
Age (years)	62.3 (11.8)	61.4 (11.7)	63.1 (12.0)	64.1 (11.8)	0.11
Female gender (%)	25.2 (*n* = 160)	25.6 (*n* = 88)	24.4 (*n* = 63)	20.5 (*n* = 9)	0.63
Hypertension (%)	54.7 (*n* = 347)	54.8 (*n* = 182)	55.4 (*n* = 143)	50 (*n* = 22)	0.79
Type 2 diabetes (%)	22.1 (*n* = 140)	22 (*n* = 73)	20.5 (*n* = 53)	31.8 (*n* = 14)	0.24
Hypercholesterolaemia (%)	54.4 (*n* = 345)	53.9 (*n* = 179)	53.5 (*n* = 138)	63.6 (*n* = 28)	0.44
Previous myocardial infarction (%)	11.2 (*n* = 71)	11.1 (*n* = 37)	11.6 (*n* = 30)	9.1 (*n* = 4)	0.88
Systolic blood pressure (mmHg)	138.6 (28.4)	138.7 (28.8)	137.8 (27.1)	142.2 (32.2)	0.91
Heart rate (beats/min)	75.8 (17.8)	75.2 (16.9)	76.3 (18.8)	76.9 (18.0)	0.94
Killip class III or IV (%)	6.5 (*n* = 41)	6.9 (*n* = 23)	5.8 (*n* = 15)	7.5 (*n* = 3)	0.85
ST-elevation in anterior leads	39.4 (*n* = 250)	39.5 (*n* = 131)	38.7 (*n* = 100)	43.2 (*n* = 19)	0.85
TIMI flow grade 3 after procedure	92.3 (*n* = 585)	93.4 (*n* = 310)	89.5 (*n* = 231)	100 (*n* = 44)	0.03
Stent implantation (%)	77 (*n* = 488)	80.7 (*n* = 268)	71.3 (*n* = 184)	81.8 (*n* = 36)	0.019
eGFR (ml/min/1.73 m^2^)	79.5 (23.2)	80.7 (24.1)	78.9 (22.4)	73.9 (19.5)	0.18
Total cholesterol (mg/dl)	195.7 (42.4)	195.3 (43.7)	195.6 (41.6)	198.5 (37.4)	0.78
HDL cholesterol (mg/dl)	43.7 (13.2)	43.6 (14)	43.6 (12)	45.3 (14.3)	0.44
LDL cholesterol (mg/dl)	128.0 (37.6)	127.9 (38.4)	127.5 (37.8)	132.1 (30.2)	0.58
Triglycerides (mg/dl)	125.2 (70)	123.7 (66.8)	128.1 (72.9)	120 (77.4)	0.8
Ejection fraction (%)	45.8 (9.5)	45.7 (9.4)	46.3 (9.5)	44.0 (10.6)	0.32
Grace risk score	149.8 (35.2)	147.1 (34.0)	152.3 (36.2)	154.6 (37.7)	0.17

Mean values with standard deviations are given, unless otherwise specified. *χ* ^2^ and Kruskal–Wallis tests were used to compare the three genotypes. eGFR-estimated glomerular filtration rate

**Table 3:** Baseline characteristics of the study group based on rs854560 genotype

Characteristic	Overall population (*N* = 634)	AA homozygotes (*N* = 296)	Heterozygotes (*N* = 269)	TT homozygotes (*N* = 69)	*p*
Age (years)	62.3 (11.8)	62.7 (11.4)	62.3 (11.8)	60.5 (13.6)	0.54
Female gender (%)	25.2 (*n* = 160)	21.6 (*n* = 64)	26.8 (*n* = 72)	34.8 (*n* = 24)	0.054
Hypertension (%)	54.7 (*n* = 347)	51.7 (*n* = 153)	58 (*n* = 156)	55.1 (*n* = 38)	0.32
Type 2 diabetes (%)	22.1 (*n* = 140)	22.3 (*n* = 66)	21.2 (*n* = 57)	24.6 (*n* = 17)	0.82
Hypercholesterolaemia (%)	54.4 (*n* = 345)	55.4 (*n* = 164)	52.4 (*n* = 141)	58 (*n* = 40)	0.63
Previous myocardial infarction (%)	11.2 (*n* = 71)	11.1 (*n* = 33)	12.3 (*n* = 33)	7.2 (*n* = 5)	0.49
Systolic blood pressure (mmHg)	138.6 (28.4)	137.7 (27.4)	138.8 (30.2)	141.4 (25.3)	0.75
Heart rate (beats/min)	75.8 (17.8)	75.4 (18.8)	76.7 (17.2)	74.1 (15.6)	0.42
Killip class III or IV (%)	6.5 (*n* = 41)	3.7 (*n* = 22)	5.9 (*n* = 16)	4.3 (*n* = 3)	0.58
ST-elevation in anterior leads	39.4 (*n* = 250)	38.9 (*n* = 115)	40.1 (*n* = 108)	39 (*n* = 27)	0.95
TIMI flow grade 3 after procedure	92.3 (*n* = 585)	91.9 (*n* = 272)	91.8 (*n* = 247)	95.6 (*n* = 66)	0.53
Stent implantation (%)	77 (*n* = 488)	76.7 (*n* = 227)	76.6 (*n* = 206)	79.7 (*n* = 55)	0.84
eGFR (ml/min/1.73 m^2^)	79.5 (23.2)	78.6 (23.2)	80.8 (23.8)	77.8 (20.2)	0.39
Total cholesterol (mg/dl)	195.7 (42.4)	197.3 (42.5)	194.1 (42.2)	195.0 (43.1)	0.81
HDL cholesterol (mg/dl)	43.7 (13.2)	44.4 (14.7)	43.6 (11.8)	41.3 (10.8)	0.31
LDL cholesterol (mg/dl)	128.0 (37.6)	130.3 (38.4)	125.4 (37.1)	128.7 (36.0)	0.46
Triglycerides (mg/dl)	125.2 (70)	127.3 (73.5)	123.3 (64.4)	124 (76.4)	0.92
Ejection fraction (%)	45.8 (9.5)	44.9 (9.4)	46.5 (9.6)	46.9 (9.4)	0.09
Grace risk score	149.8 (35.2)	152.1 (38.4)	148.6 (32.7)	144.3 (30.1)	0.38

Mean values with standard deviations are given, unless otherwise specified. *χ* ^2^ and Kruskal–Wallis tests were used to compare the genotypes. eGFR-estimated glomerular filtration rate

### Survival analysis

**Fig. 1:**
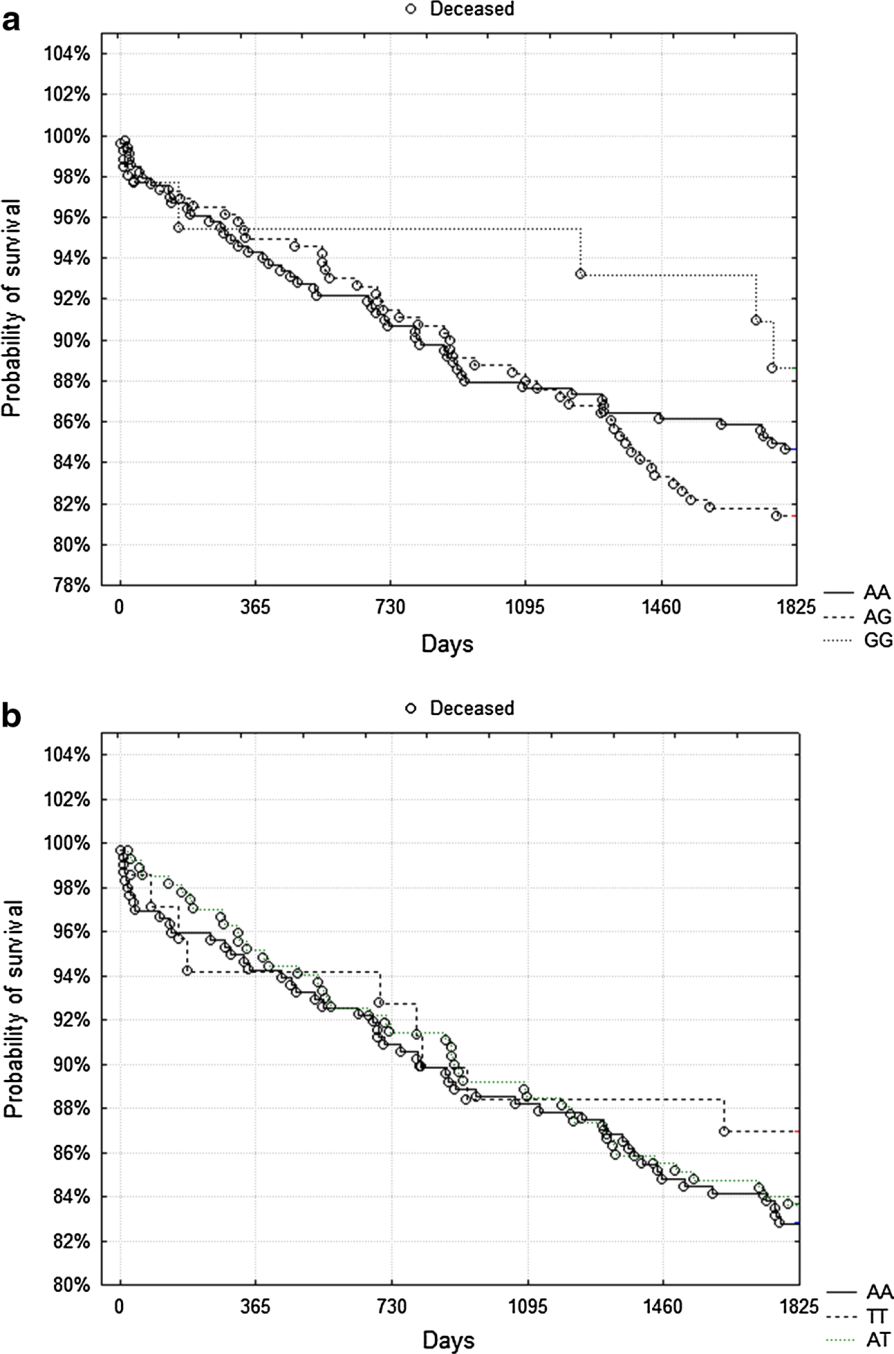
**a** Kaplan–Meier surviving curves for specific genotypes of rs662 polymorphism and 5-year mortality (*p* = 0.4, log-rank test). **b** Kaplan–Meier surviving curves for specific genotypes of rs854560 polymorphism and 5-year mortality (*p* = 0.73, log-rank test)

**Fig. 2:**
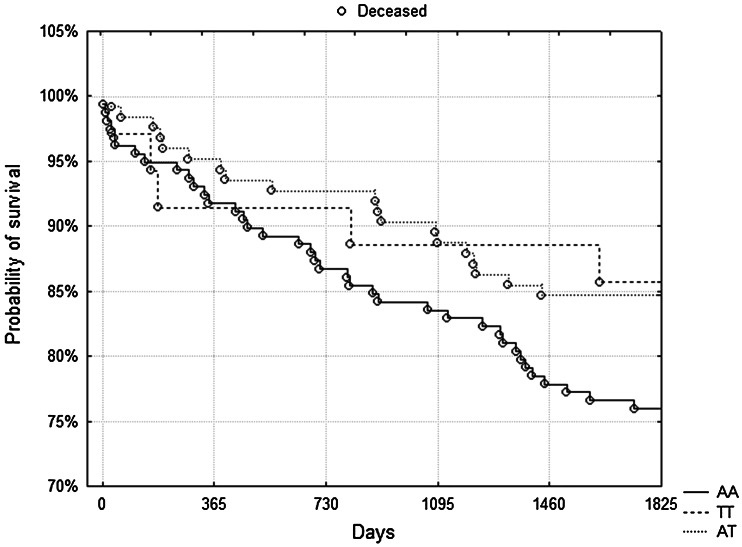
Subgroup of patients with eGFR below median value. Kaplan-Meier surviving curves for rs854560 polymorphism and 5-year mortality. AA homozygotes had significantly lower probability of survival compared to other genotypes (*p* = 0.047, log-rank test)

During 5-year follow-up, 104 patients died (16.4 %). We observed no association between either genotype and survival. Figure 1 shows Kaplan–Meier surviving curves for specific genotypes of rs662 and rs854560 polymorphisms and 5-year mortality (*p* = 0.4 and *p* = 0.73, log-rank test). However, in a subgroup of patients with eGFR below median value (78.6 ml/min/1.73 m2, *n* = 317) the rs854560 AA homozygotes had a significantly lower probability of survival compared to other genotypes (Fig. 2, *p* = 0.047, log-rank test). There died 38 out of 158 AA homozygotes (24 %) and 24 out of 159 T-allele carriers (15.1 %, *p* = 0.044, *χ* ^2^ test). In the case of the rs662 SNP there was no influence on survival depending on eGFR (data not shown).

Table 4 presents parameters associated with 5-year survival in a subgroup of patients with eGFR below median value (univariate and multivariate analysis- Cox proportional hazard model). The rs854560 AA genotype was one of the variables associated significantly with adverse 5-year survival (OR = 1.66, 95 % CI 1.0007–2.78, *p* = 0.049).

**Table 4:** A univariate analysis for 5-year mortality in a subgroup of patients with eGFR below median value. For all parameters baseline values are shown

Variable	Odds ratio (95 % CI)	*p*
Univariate analysis		
Age (years)	1.05 (1.02–1.08)	0.0002
Type 2 diabetes	2.0 (1.2–3.3)	0.009
Arterial hypertension	1.5 (0.84–2.5)	0.17
Systolic blood pressure (mmHg)	0.99 (0.98–0.999)	0.04
Heart rate (beats/min)	1.0 (0.98–1.01)	0.99
Killip class	1.9 (1.5–2.4)	<0.0001
Total cholesterol (mg/dl)	0.99 (0.98–0.997)	0.008
HDL cholesterol (mg/dl)	0.99 (0.97–1.01)	0.37
LDL cholesterol (mg/dl)	0.99 (0.98–1.0)	0.056
Triglycerides (mg/dl)	0.996 (0.992–1.001)	0.17
Ejection fraction (%)	0.95 (0.93–0.97)	0.0001
TIMI 3 flow after invasive procedure	0.42 (0.22–0.86)	0.01
Grace risk score	1.02 (1.012–1.02)	<0.0001
Rs854560 AA genotype	1.67 (1.0–2.8)	0.049
Rs662 AA genotype	0.88 (0.53–1.45)	0.63

Multivariate analysis	*χ* ^2^ = 40.9	
Age (years)	1.045 (1.017–1.07)	0.0012
Killip class	1.8 (1.4–2.3)	<0.0001
Type 2 diabetes	1.79 (1.07–3.0)	0.025

## Discussion

Our study showed worse 5-year survival of the AA homozygotes of the rs854560 polymorphism, but only in a subgroup of patients with eGFR below median value. This phenomenon may be explained by decreased PON1 activity in the AA homozygotes of rs854560 SNP [7]. This results in the diminished antioxidant activity of HDL cholesterol, an increase in oxidative stress and endothelial dysfunction [6, 17], including decreased NO production [6].

Impaired renal function is associated with poor long-term outcome of patients with myocardial infarction [18–20]. One of the underlying mechanisms could be decreased HDL antioxidant activity [4], which would additionally augment the effect of rs854560 AA homozygosity and may trigger its phenotypic effect in terms of survival.

This observation was not confirmed for the rs662 genotype; however, the two investigated polymorphisms are in weak linkage disequilibrium. We also did not replicate associations between PON1 polymorphisms and either myocardial infarction or lipid profile. In general, this study was not designed for this purpose: however, the lack of such a link in a population of 634 patients undermines the clinical significance of this association.

Previous surveys concerning the investigated SNPs and coronary artery disease gave ambiguous results [3, 10–13]. Generally, in the case of rs662 polymorphism, an AA genotype associated with lower PON1 activity is considered as high-risk [3]. However, it has been reported that PON1 activity in AA homozygotes decreases with advancing age when compared with other genotypes [3]. It is noteworthy that those authors showing G allele as a high-risk, reported the association with myocardial infarction only in relatively early-onset cases: <50 years [12] or <60 years [11]. Genotype-phenotype correlation might differ between populations due to multiform interactions based on genetic background and other clinical features. Therefore genetic studies should be validated in specific populations.

The GG genotype of the rs662 SNP was previously reported to be associated with a favorable lipid profile in the general population (lower total cholesterol, LDL fraction, triglycerides and apolipoprotein B levels, higher HDL cholesterol level) [8]. This result was not replicated in our study group of patients with myocardial infarction. In general, in studies enrolling patients with coronary artery disease, cholesterol status is strongly influenced by statin treatment. Such patients are not an appropriate group for revision of the influence of genetic factors on lipids. Interestingly, we observed in our study the so-called “cholesterol paradox”. Lower total cholesterol levels were associated with an adverse outcome. This phenomenon has previously been reported in some studies including patients with myocardial infarction [15, 16, 21–24]. In our case, this finding could have potentially increased the favorable effect of the GG genotype on survival, if such was observed.

The next parameter that potentially influences survival is oxidative stress. It has been shown that the AA genotype is associated with the highest oxidative stress markers levels and subsequent all-cause mortality in a group of patients undergoing coronary angiography [25]. On the other hand, tumor necrosis factor alpha−one of the systemic inflammation markers− was shown to be significantly higher in GG homozygotes [26].

PON1 has an impact on clopidogrel absorption and activation [27]. This phenomenon was not confirmed in vivo and has no clinical impact. All these findings show complexity of the link between PON1 SNPs and cardiovascular disease or subsequent mortality. They also may explain hypothetically ambiguous results of previous studies.

### Limitations of the study

We are fully aware of the limitations of the study. The analysis was performed retrospectively (however the data was collected in a prospective manner). Next, the number of patients included is relatively small. Large study groups enable finding associations of very small effect sizes, but these frequently have no real clinical impact. Investigating large effects are more promising for clinical practice, as more cost-effective.

## Conclusions

The AA genotype of the rs854560 SNPs of the PON1 gene was associated with increased 5-year mortality of patients with STEMI treated invasively, but only under condition of eGFR decreased below median value.
